# Exploring the cerebral substrate of voice perception in primate brains

**DOI:** 10.1098/rstb.2018.0386

**Published:** 2019-11-18

**Authors:** Clémentine Bodin, Pascal Belin

**Affiliations:** 1 Institut de Neurosciences de la Timone, UMR 7289 Centre National de la Recherche Scientifique and Aix-Marseille Université, Marseille, France; 2 Département de Psychologie, Université de Montréal, Montréal, Canada

**Keywords:** language evolution, primates, voice patch system, voice perception, comparative neuroimaging

## Abstract

One can consider human language to be the Swiss army knife of the vast domain of animal communication. There is now growing evidence suggesting that this technology may have emerged from already operational material instead of being a sudden innovation. Sharing ideas and thoughts with conspecifics via language constitutes an amazing ability, but what value would it hold if our conspecifics were not first detected and recognized? Conspecific voice (CV) perception is fundamental to communication and widely shared across the animal kingdom. Two questions that arise then are: is this apparently shared ability reflected in common cerebral substrate? And, how has this substrate evolved? The paper addresses these questions by examining studies on the cerebral basis of CV perception in humans' closest relatives, non-human primates. Neuroimaging studies, in particular, suggest the existence of a ‘voice patch system’, a network of interconnected cortical areas that can provide a common template for the cerebral processing of CV in primates.

This article is part of the theme issue ‘What can animal communication teach us about human language?’

## 1. Introduction

The question of language evolution is certainly one of the most challenging questions of our times. Since Charles Darwin's pioneering ideas almost two centuries ago [1], extensive research now supports a scenario where language has been gradually shaped from animal precursors instead of a sudden and recent emergence in the human lineage. Rather than a single encapsulated entity, language is considered as a set of cognitive components that may or may not be present at varying degrees in other animal species [2]. By building cognitive phylogenies of these components, we can thus capture crucial information about the emergence of language and the factors that influenced it, which could have been neglected if considered as a whole [3]. Recent breakthroughs in the field of vocal communication highlighted several of these components in non-human primates (NHP), including vocal learning [4,5], a rudimentary form of grammar [6–10], together with sequence learning abilities [11–13] and also the presence of a semantic content [14–16] and intentionally in their vocalizations [17].

What emerges as a connecting thread across these different components of vocal communication is the perception of voice, i.e. the processing of information carried by the caller's voice. This fundamental ability is a key element of communication for a wide range of species and is therefore particularly appropriate to bridge the gap between animal communication and human language. In particular, an efficient processing of conspecific vocal signals is crucial in a number of situations such as competition for territory, parental care, reproduction or predator avoidance. From these essential behaviours arises the need to infer the vocalizer's size, age, sex, group membership, individual identity or inner state, to adjust behaviour accordingly. This non-verbal content of voice can be distinguished from speech in human language and is also present in other animal vocal signals. Although vocalizations exhibit a certain specificity due to species-specific ecological constraints, the perceptual mechanisms involved in the processing of non-verbal information are probably more conserved. Since neither behaviour nor its brain substrate can be directly investigated from fossils, comparing humans to the closest extant species, NHP, can be used to infer the recent evolution of voice perception before the emergence of language. Here, particular attention is given to what is shared across primates rather than what separates them.

In the present paper, voice perception will refer primarily to the processing of information in conspecific vocalizations (CV) despite evidence that primates are also able to extract information from heterospecific vocalizations [18–22]. CV perception is assumed to include several processing stages that are organized in a similar way to those employed to extract information from faces [23,24], from distinguishing CV among non-CV sounds (initial ‘structural encoding’ stage) to processing different types of information contained in CVs (e.g. species, identity, gender, emotional state, etc.) in interacting but segregated functional pathways. Here, a particular interest will be given to the speaker/caller identity recognition as involving high-level processing stages in both systems. Since it is only in humans that voice perception abilities also include speech perception, this particular type of CV information will not be discussed.

We start by summarizing behavioural evidence of voice perception in primate species ranging from New World monkeys to apes (marmoset, macaque, chimpanzee and human). These species were selected based on the available neuroimaging literature on the cerebral basis of voice perception, developed in the second part of the paper. From this evidence, we develop our hypothesis of a conserved ‘voice patch’ system in primates dedicated to process CV information. This network of voice-sensitive areas can be compared to the face-processing system of the visual cortex [25–28]. More generally, we assume that cross-species similarities constitute evidence for homologous mechanisms inherited from a common ancestor and a gradual evolution of voice perception [29,30]. In the final section, we suggest future directions for comparative research on voice perception.

## 2. Behavioural evidence of conspecific voice perception

**Figure 1.**
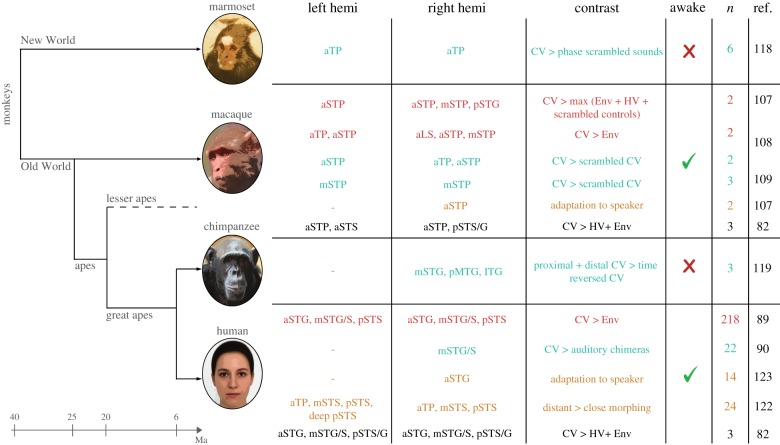
Neuroimaging evidence of the temporal lobe regions showing sensitivity to conspecific voice (CV) in primates. A simplified phylogenetic tree of the species of interest is represented on the left (common marmoset, rhesus macaque, chimpanzee and human). On the right side, the table summarizes the regions found in neuroimaging studies (references in the last column) for the left and right hemispheres separately. The other columns indicate the main differences in the experimental procedures used in these studies (contrast of interest, anaesthesia and number of individuals). Three main categories of contrasts emerge: CV > non-CV (red), CV > acoustic controls (green) and identity sensitivity (orange). In black, we added recent results that we obtained in a comparative study between human and macaques. a, m, p, anterior, middle, posterior; Env, environmental sounds; HV, heterospecific voice; ITG, inferior temporal gyrus; LS, lateral sulcus; MTG, middle temporal gyrus; Ma, million years ago; STP, superior temporal plane; STG/S superior temporal gyrus/sulcus; TP, temporal pole.

The primate auditory channel, together with vision, evolved as the main communication mode relative to the olfactory and chemical channels predominant in other animal species. Marmosets, macaques and chimpanzees diverge from the human lineage about 40 and 25 and 6 Ma, respectively (figure 1, left). These species have complex, albeit fairly different, social behaviours that can be regulated using a specific set of conspecific vocalizations. Humans, in particular, have remarkable abilities to extract verbal and also non-verbal information from CV, such as identity [31–33], gender [34] or personality [35]. This process is already operational in early infancy to recognize parents' voice [36–38] and the emotional content of voice [39]. Extraction of caller information is known to reflect the source-filter theory of voice production [40] where acoustic cues derived both from the larynx (fundamental frequency, f0) and the upper vocal tract (mostly formants or vocal tract resonances) are involved [31,41,42]. Surprisingly, it is only quite recently that a behavioural advantage of voice detection has been experimentally demonstrated in humans: both categorization and detection have been seen to improve when human voices (CV) are the targets compared to non-vocal sounds [43–45].

In macaques, belonging to the Old World monkeys, vocalizations are mainly produced to regulate and coordinate group activities using a rich call repertoire divided into a dozen of classes according to the social context and the motivational state [46–50]. One early series of studies in Japanese macaques employing category identification tasks showed that they can discriminate different CV from the ‘coo’ class of their repertoire and in a more efficient way than the compared species [51–55]. Nevertheless, further research on monkeys not intensively trained for this discrimination suggests a gradual transition through the different CV within the ‘coo’ and ‘screams’ classes rather than discrete boundaries [49,55–57]. In addition, the intrinsic variability of each class is not equal [58] and, hence, potentially conveys distinct information. As in humans, there is clear behavioural evidence that macaques can use identity information from CV [47,59–61]. They seem to rely on formant frequency information, in view of their ability to perceive formant frequency changes in playback trials [62] and associate these changes to differences in perceived body size [63,64]. Although macaques show moderate sexual dimorphism in body size, it is not clear whether macaques can recognize gender from CV.

Recently, there has been a renewed interest in New World monkeys such as marmosets that form a group of social and territorial species living in the upper canopy of South American forests. As highly vocal animals, they are in almost constant vocal communication, even in captivity [65,66], and their repertoire is now well-characterized acoustically [67–69]. This includes multiple vocalization types, from both simple to compound calls composed of sequences of simple calls [67]. This sequential production is highly variable in the temporal domain and can be modulated by the emotional state [70]. However, the processing of complex sequences remains limited in these species [71]. One long-distance contact call, the ‘phee call’, is produced by visually separated congeners in a reciprocal exchange known as ‘antiphonal calling’ [72]. This call contains potentially significant information on the caller's identity [68] or gender [73]. Recent evidence demonstrated that marmosets can process identity from these calls. The ‘Virtual Monkey’ approach, an automated playback technique, exploited the antiphonal calling behaviour: changes in the identity of synthetic phees were followed by changes in the frequency and latency of antiphonal calling by the subject, demonstrating identity discrimination [74]. Others have shown that changes in caller identity during playback can induce exploratory behaviour in marmosets [75].

Behavioural evidence of CV perception in great apes is much less documented, especially because they are more rarely studied in the laboratory. Chimpanzee vocalizations have been described in association with their facial displays as graded among acoustically defined call categories from simple, well defined, categories to more blended ones according to the internal motivations [76]. Considered separately, a large proportion of compound calls have been recorded, some of them exhibiting a different function than their simple counterpart [77]. A limited number of studies suggest that identity can be processed from chimpanzee CV. One of them found markers of individuality in the acoustics of one long-range call [78].

Two others employed vocal-to-facial matching tasks and reported correct identification of the caller from both long-range and short-range calls [79,80]. In contrast with this sparse literature on CV perception, higher-level properties of communication like intentionality and meaning have been described for chimpanzee vocalizations [15,17].

Thus, the evidence presented in this section indicates that CV of other NHP convey relevant information to their social interactions, as does non-verbal information in the human voice. The perceptual mechanisms involved in CV processing could then be conserved to some extent due to selective constraints inherent to this social life. Thanks to the recent advances in non-invasive neuroimaging techniques, the cerebral mechanisms of CV processing in primates can now be more precisely examined and compared with those of humans.

## 3. Cerebral evidence of conspecific voice processing in the temporal lobe

### (a) Clarifying where and what we are looking at

**Figure 2.**
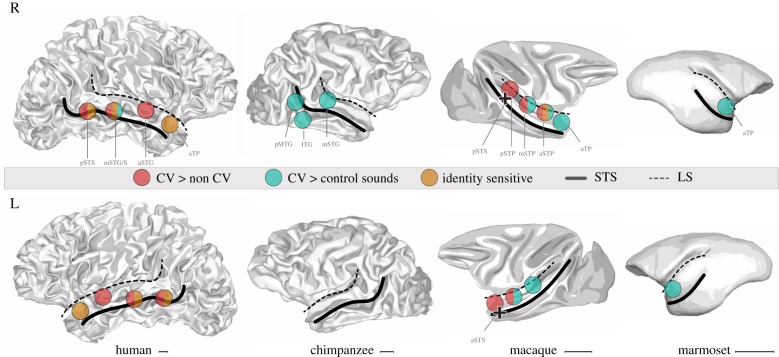
Organization of the conspecific voice (CV) patches along the temporal lobe in primates. The location of the regions is based on the selected neuroimaging studies in figure 1. Coloured spheres represent the location of a region more sensitive to CV than: non-CV (red), acoustic controls (green), a particular sensitivity to identity (orange) or to several of these contrasts (mixed colour). Black crosses illustrate the position of the newly identified CV-sensitive STS sites [82]. The regions are represented on white matter surfaces to reveal the inner part of the folds. The brain surfaces were modified from personal data for human, one individual image of the National Chimpanzee Brain Resource (NS092988) for chimpanzees, the NMT atlas white matter surface for macaques [83] and the segmented Brain/MINDS atlas for marmosets [84]. The black bar indicates 1 cm scale. L, left hemisphere; R, right hemisphere; a, m, p, anterior, middle, posterior; CV, conspecific voice; ITG, inferior temporal gyrus; LS, lateral sulcus; MTG, middle temporal gyrus; STP, superior temporal plane; STG/S, superior temporal gyrus/sulcus; TP, temporal pole.

In primates, the anatomical organization of the auditory cortex reflects a functional hierarchy where information flows through primary regions (the ‘core’), secondary regions (‘belt’ to ‘parabelt’) and auditory related fields, extending principally from the lateral sulcus (LS) to the superior temporal sulcus and to the extra-temporal regions [81]. The primary auditory cortex is located in Heschl's gyrus in humans but is deeply hidden in the LS, behind the parietal opercula, in monkeys. Different cytoarchitectonic parcellations of the auditory cortex have given rise to various nomenclatures. This potential source of confusion makes the interspecies comparison difficult to assess. Here, we chose to report the data from the literature (figures 1 and 2) based on simple morphological references. The medial-lateral axis is represented in order by the LS, the superior temporal plane (STP), the superior temporal gyrus (STG) and the STS. The positions on the rostral to caudal axis are noted as anterior (a), middle (m) and posterior (p). Another potential source of confusion in the cross-species comparison concerns the nature of the contrast used to highlight a CV sensitivity. Two principal methods exist: the first (CV versus non-CV) compares CV with categories of complex sounds such as heterospecific (HV) or environmental (Env) sounds; while the second (CV versus control sounds) compares CV with acoustically matched control sounds for which a specific set of acoustic parameters are kept unchanged (e.g. temporal envelop in phase-scrambled sounds). A third type of contrast specifically examines the sensitivity to identity information. Figure 1 summarizes the approximate anatomical location of contrasts reported in key selected studies for each species, classified into three main categories: CV > non-CV (red), CV > acoustic controls (green) and sensitivity to speaker/caller identity (orange). Each selected study is further developed in the following section.

### (b) Neuroimaging evidence of conspecific voice perception

The human cerebral substrate for voice perception is centred on secondary auditory cortical regions located bilaterally along the superior temporal gyrus (STG) and sulcus (STS). These ‘temporal voice areas' (TVAs) show greater fMRI signal in response to vocal sounds, whether they contain speech or not, compared to other categories of non-vocal sounds such as environmental sounds, amplitude-modulated noise [85–87], or to hetero-specific vocalizations (HV) [88]. Although their exact anatomical location in the temporal lobe varies considerably across individuals, a cluster analysis of voice-sensitivity peaks in several hundred subjects highlighted an organization in three ‘voice patches’ along STG/STS bilaterally (TVAa, TVAm and TVAp) [89] (figure 1). To test their selectivity, Agus *et al*. [90] used ‘auditory chimeras’ that matched vocal sounds for a large subsets of acoustical features and showed that the right TVAs (middle STG/STS) were preferentially activated only for the natural human voice. Very little is known about the cortical anatomy underlying the TVAs, on average centred bilaterally around the deep portion of the STS but exhibiting an important variability at the individual level [91]. Functional connectivity investigations have highlighted both intra- and inter-hemispheric connections between the different TVAs during passive voice listening [92] or a voice recognition task [87]. The three patches of the right STS were also shown to be structurally connected to each other [93]. Thus, while it seems clear that vocal information flows through the voice areas, little is known about their individual roles to date. Nevertheless, a growing body of literature indicates that they are recruited differently to process identity (reviewed in [27]).

As for behavioural evidence, our knowledge of the cerebral substrate for voice perception in NHPs mainly comes from the studies on rhesus macaques and marmosets, which are standard animal models in neuroscience. Nevertheless, pioneering studies were initially conducted in the auditory cortex of the squirrel monkey revealing CV sensitivity at the neuronal scale [94,95]. Electrophysiological recordings in awake macaques evidenced neurons in belt and parabelt areas that show a strong sensitivity to CVs [96–99] with latencies and selectivity increasing in the caudo-rostral direction towards the temporal pole [100,101]. Conversely, a strong sensitivity to CVs was found in the core areas of freely moving marmosets using electrophysiology [102–104]. Despite the crucial information provided by electrophysiology, direct comparison between species remains difficult because electrophysiological recordings in humans are mostly extracranial and, therefore, at low-spatial resolution. Recent advances in functional imaging hold much promise as they allow scanning of awake animals using protocols comparable to those of humans [105,106].

Petkov *et al*. [107] was the first fMRI study revealing macaque voice patches with responses analogous to the human TVAs, i.e. areas with significantly stronger response to macaque CV than other categories of environmental (CV versus non-CV) or control sounds (CV versus control sounds). Using an auditory cortex parcellation, a bilateral but mostly rightward activity of the anterior STP was found together with two clusters in the right middle STP (close to primary auditory cortex) and posterior STG. A right anterior patch was observed in the same location in two individuals and was still observed in anaesthetized monkeys, removing the possible effect of attention to sounds. It was then targeted in a subsequent single-cell recording electrophysiology study that revealed ‘voice cells’ in that anterior voice patch [97]. Surprisingly, no further consideration was given to the other voice patches.

Another study by Ortiz Rios *et al*. [108] also reported CV selectivity: compared to environmental sounds (CV versus non-CV) CV activated more bilateral anterior STP and right middle STP in addition to more anterior regions of the temporal pole. However, they recruited only the anterior patches when compared with their scrambled version (CV versus control sounds), altered spectrally and temporally.

Joly *et al*. [109] performed a pioneering comparative study in which human (*n* = 20) and macaque (*n* = 3) subjects were scanned in the same scanner while exposed to the same stimuli, including vocalizations of both species. Areas along the middle STP, close to A1, showed greater response to CV compared to their scrambled controls, but not compared to human vocalizations.

It has been suggested that the position of the anterior voice area described in Petkov *et al*. [107] is different from what was expected from human data [30,110]. Yet this comparison only focused on the most anterior patch observed in two macaques, leaving the possibility that the other, not yet examined, voice patches could be more similar to those of humans. In fact, taking into account all relevant studies and combining the different contrasts and nomenclatures used, several voice patches clearly emerge along the STG, in both hemispheres of the macaque brain. In a recent fMRI study, we scanned three awake macaques and three humans using a similar paradigm of passive auditory listening [82]. We found that CV elicited more activity in the bilateral anterior STP and the right posterior STG than environmental and heterospecific sounds, replicating earlier findings. For the first time, however, we found that the CV versus non-CV contrast also recruited the STS in its left anterior and right posterior portions (black crosses in figure 2), as in humans.

These novel results support the existence of several voice patches in monkeys; they also point towards a recruitment of STS domains. Indeed, this region has often been neglected as a classified multisensory region only. However, neuronal recordings already reported unimodal auditory responses in macaque STS [111–113]. Using face and voice stimuli, the anterior STS was shown to contain a more balanced proportion of auditory and visual unimodal neurons than the anterior STP; however, multisensory interaction was found to be equally prominent in both regions [113].

The past 5 years have seen increasingly rapid advances in the field of marmoset brain imaging [114–117], particularly as its small size is compatible with highfield (7T) rodent MRI allowing for higher signal and spatial resolution. Remarkably, a recent fMRI study in anaesthetized marmosets (*n* = 6) [118] revealed a gradient of sensitivity to CV along a caudal-ventral axis [118], with areas of high selectivity (here compared to scrambled controls) in the most anterior parts of the temporal lobe bilaterally. Such CV sensitivity accords with the anterior patches previously described in other species and with the neuronal gradient of selectivity of the macaque STP [101]. However, this is inconsistent with the other patches and previous reports of CV sensitivity in the marmoset primary cortex [102–104], located more dorsally. Although speculative, it is still possible that anaesthesia may lower the signal strength and allow only the regions that are least sensitive to the level of vigilance to be seen. One PET (positron emission tomography) study started to fill the gap between humans and monkeys by studying CV sensitivity in anaesthetized chimpanzees [119]. By grouping both proximal (short-range) and distal (long-range) categories of calls in contrast to their time-reversed controls (CV versus controls), the activity was lateralized on the right posterior temporal lobe, with peaks extending from the superior to the inferior temporal gyrus. However, this posterior activity was mainly driven by proximal calls and an important variability seemed to exist across conditions and individuals. Three methodological factors can have induced such variability and could explain the divergence with other species. First, PET acquisition was necessarily delayed after the auditory listening task during which the radioactive tracer is injected and this delay may have varied between individuals. Second, the auditory listening task was performed on freely moving animals without control of interaural differences. Finally, time-reversed controls may have involved different processes than other matched controls described in figure 1. For instance, in macaques, temporal inversion was shown to induce distinct behavioural responses depending on the acoustical symmetry of the call [120,121]. Hence, although a promising investigation, there is abundant room for further progress in determining the localization of voice areas in chimpanzees and it would be premature to interpret the observed lateralized activity.

Neuroimaging studies in the different primate species mentioned above are summarized in figure 2, which illustrates our current knowledge of the putative location of voice patches in the temporal lobe of humans, chimpanzees, macaques and marmosets.

### (c) Identity processing

Speaker identity processing in humans involves both temporal and prefrontal regions with strong right-hemispheric lateralization [122,123]. The most anterior voice-sensitive region of the right temporal lobe (right TVAa) in particular shows adaptation to speaker identity, i.e. smaller response to syllables spoken by a single speaker than to syllables spoken by multiple speakers [123]. A similar adaptation procedure evidenced a sensitivity to gender in this region [124]. Subsequently, Andics *et al*. [122] showed that bilateral TVAs are recruited by contrasting close versus distant identities morphed along an acoustic continuum. Nevertheless, all clusters in the right hemisphere, but only the deep left STS, were positively correlated with recognition performance. This is in line with the idea that unfamiliar voices are coded in the TVAs in a multidimensional acoustical ‘voice-space’ [31,125]. In particular, voices acoustically close to their (own-gender) average prototype elicit smaller TVA activity than more distinctive, acoustically dissimilar voices as a ‘norm-based’ coding [125]. It is worth noting that familiar and unfamiliar voice may be processed through dissociate pathways and thus make the prototype model more complex than expected (as reviewed in [33]). Andics *et al*. [122] also described interesting adaptation effects (response reduction to stimuli perceived as similar) along the STS axis: a short-term acoustic adaptation in the bilateral middle/posterior STS but a longer-term identity effect in the anterior temporal poles and the deep posterior STS. This may suggest that CV is primarily processed acoustically in middle and posterior TVAs then addressed to the anterior patches to extract identity-relevant information. A preponderant involvement of the right anterior region in that processing is suggested by adaptation mechanisms [123] and information-decoding procedures [122,123]. Contrastingly, the role of the right posterior region is nuanced by two contradictory lesion studies: from a cohort of patients, one classified it as an obligatory structure for voice-identity recognition [126], whereas another case study reported no effect on voice perception or identity recognition after a complete right pSTS resection [127]. As TVAs are functionally connected to each other but also to frontal regions during voice perception [92], it would be interesting to explore if compensatory mechanisms exist after such a lesion.

In macaques, the right anterior voice area described earlier exhibits the same speaker adaptation response to that observed in the human right anterior temporal lobe: greater response to CV from different individuals than CV from a single individual [107]. Some of the voice cells in that region also show some degree of caller selectivity, differentiating between individuals more than call type [113]. Advances in the processing of identity in these monkeys have been pushed forward by the discovery of multisensory regions integrating vocal and facial information and converging toward the temporal pole (reviewed in [128]; see section *Parallel with the face-processing system*).

Despite behavioural evidence that identity is relevant to marmosets, cerebral evidence of such processing is not obvious. In a recent PET study [75], extra-temporal regions were found to be associated with the perception of phee calls from a single subject compared to multiple subjects' stimuli. However, an adaptation effect could have been expected in their CV-sensitive anterior temporal poles [118] by contrasting these two conditions. To our knowledge, there is no experimental evidence to the neural coding of caller identity in chimpanzees.

### (d) Conspecific voice perception in extra-temporal regions

Neuroimaging studies also revealed extra-temporal regions, sensitive—although less consistently—to CV. Three bilateral patches were identified in the human frontal cortex as the ‘frontal voice areas’, more sensitive to voice than non-CV stimuli [92]. Especially, voice recognition performance was related to the functional connectivity into this fronto-temporal network in the right hemisphere [92]. The previously cited literature in macaques (figure 1) also reported CV sensitivity in parietal and ventro-lateral prefrontal cortex together with higher-level visual areas. In particular, electrophysiological recordings provide clear evidence that prefrontal cortex contains CV-sensitive neuronal populations; however, it is not yet clear whether they constitute higher-level areas in voice processing than those of the temporal lobe (reviewed in [98]). A study combining fMRI with neuronal microsimulation suggests that the level of processing of temporal areas cannot predict their effective connectivity with frontal areas [129]. Nevertheless, it is highly possible that anterior temporal and prefrontal cortices collaborate during CV processing as part of the same ventral pathway for complex sounds processing [130,131].

## 4. A voice patch system hypothesis

The literature overviewed here support the notion of a conserved system for the perception of CV in primates. Several discrete areas along the primate STG exhibit a specific sensitivity to voice compared to other categories of natural sounds or matched controls. These areas extend from belt to auditory related fields up to polar regions of the temporal lobe [81]. Future work is needed to provide a generic model linking the diverse results gathered so far in a coherent picture. First, it becomes essential to better determine the role of STS in the processing of CV in monkeys. Too long considered as only multimodal, this view is challenged by the presence of unimodal auditory neurons [111–113] and now by new data [82]. Second, additional neuroimaging investigations should be carried out in chimpanzees and marmoset to counterbalance the increasing amount of evidence in humans and macaques.

We further discuss three points in relation to the hypothesis of a potentially conserved voice patch system: (i) the voice patch system [132] is organized into a network of interconnected voice patches comparable to those of the face-processing system of visual cortex [25–28]; (ii) supporting an ecologically relevant ability in primates, it could be part of a broader social network in their brain; (iii) speech emerged from primitive roots including the voice patch system.

### (a) Analogies and interactions with the face-processing system

Full characterization of the voice patch system could allow further testing of the hypothesis of similar coding strategies for processing face and voice [24], converging across sensory modalities to extract, for example, identity information. Studies in humans, macaques and, more recently, marmosets together demonstrate the existence of a system of discrete, interconnected face-sensitive areas containing ‘face cells’ and supporting a series of increasingly abstract (identity-invariant) face representations [25–28,133]. The overall arrangement of face patches and their approximate distribution in the occipito-temporal cortex appears quite similar across species, although there is an overall shift of areas ventrally from the STS in humans compared to macaques [134]. This shift also seems to apply to a lesser extent to voice areas, with areas more deeply located in the STP in monkeys, but mostly around the STS in humans. Although we still do not know if there is a vocal equivalent of the view-point invariance gradient observed in the face network, adaptation and multivariate paradigms [107,123,135] suggest that invariant, word-independent, vocal identity information is mainly processed in anterior temporal regions in both humans and monkeys. Further work would determine whether NHP represent different callers in a measurable ‘voice identity space’ similar to humans and if they rely on norm-based coding strategies. From the face processing literature, behavioural evidence indicates that chimpanzees but not macaques rely on a norm-based coding of facial identity [136], whereas neuronal recordings suggest that this coding is also present in macaques [137]. As for the face patches, structural [93] and functional connections [92] were also found between the different voice patches, meaning that both systems could constitute an interconnected network.

Yet, in contrast to the notion of a conserved face-patch system across primate brains, behavioural studies report striking differences between great apes and monkeys in the way they encode faces [138]. An investigation of the cerebral basis of face processing in chimpanzees identified bilateral face-sensitive regions mostly localized around orbitofrontal and posterior STS areas [139], the latter being close to the voice-sensitive regions in this species (figure 2). Although new investigations are required to confirm this result, it indicates some inconsistencies across primates as observed for voice in our case (figure 2). Perhaps the key lies in the way voice and face processing systems interact with each other. Several analogies between the two systems have been established from behaviour to cerebral bases between humans and monkeys [24,128]. The most direct evidence of their interaction is probably their connectivity. Blank *et al*. [93] reported direct structural connections between voice and ventral face areas in humans, and von Kriegstein *et al*. [140] a functional coupling of these regions during familiar speaker recognition. Both findings indicate a multimodal integration for identity recognition. But where could this integration take place? A model largely inspired by monkey data suggests a multimodal interaction that would converge towards the macaque temporal pole region to extract identity information [128], which is also supported by electrophysiological recordings and neuroimaging studies (figure 2). Extracting identity from voice in humans was previously shown to occur in the right anterior STS and over a large part of the TVAs by adaptation and morphing paradigms respectively [122,123] (figure 1). However, extracting identity from both face and voice could engage the right posterior STS in a modality general representation (e.g. [141–143]) by an audio-visual integration phenomenon (e.g. [141,144]), although others claim that this multimodal association is preferentially processed in inferior parietal areas [126]. Hence two identity processing streams emerge from the human literature. On the one hand, voice is processed along the STG/S antero-posterior axis using specific circuits depending on its familiarity (reviewed in [33]) to be fully recognized and stored [122] in the anterior temporal lobe. On the other hand, a multimodal pathway involving the posterior STS and inferior parietal areas integrates information carried by the face and voice. The latter lacked evidence in monkeys and could be inextricably linked to the social nature of the transmitted information such as emotions [144] and social stimuli perception [145,146] in humans.

Hence, although face and voice processing systems would be conserved in primates, their functional interactions may differ between species. Strikingly, the temporo-parietal junction (TPJ) has undergone an increasing cortical expansion from New World monkeys to great apes [147], along with a major restructuring of its anatomical and functional organization [148]. This expansion may have influenced a ventral shift of both the voice- and the face-patch systems from monkeys to humans and favoured the emergence of a new functional pathway combining multimodal social information in and beyond the pSTS in humans [148].

### (b) Integrated into a broader social network?

From a larger perspective, we assume that the vocal exchanges of information essential to the primates' social life may have shaped their voice processing system in a similar way. As an example, similar encoding strategies in their vocalizations [149] could allow a generalization across call categories but also across the vocal repertoires of other species [18–22]. To what extent has social life been able to determine these similarities in voice processing among primates? The social networks involved in the perception of interacting faces and in orofacial movement have been recently described in macaques, highlighting similarities with the speech-production system in humans [150]. At least two of these networks also interact with voice in humans and monkeys: the face-processing system (see previous section) and the lateral prefrontal cortex (e.g. [80,86,92,117]). In daily life, faces, voices and orofacial movements are in constant interaction during communication, which can suggest that the CV-processing system is also part of a broader social network in the primate brain. Importantly, whereas the cerebral substrates (see previous sections) and coding strategies [149] appear relatively conserved through evolution, voice and face processing systems are still permeable to the early social environment. For example, a study on face-deprived infant macaques [151] demonstrated that exposure to faces was necessary for the emergence of the face patches and a behavioural interest in those stimuli. In marmosets, parental feedback can affect vocal development and the acoustic structure of the infant's calls [152]. In humans, voice areas become functional between four and seven months of age [153], while a behavioural tuning to speech over heterospecific vocalizations seems to arise at three months of age [154]. The influence of the auditory environment on the development of CV-processing networks in primates remains to be investigated experimentally.

### (c) How did speech processing integrate into the voice patch system?

Evidencing similarities between human and NHP can help bridge the gap between animal communication and human language. However, this purpose is made challenging by the tight relationship between speech and voice in humans. The identity- and speech-processing pathways of voice perception constantly interact, in no small part because the same acoustical cues (formant frequencies) allow perceiving both what is being said [155] and who is speaking [156]. In the brain, speech stimuli elicit higher activity in the temporal voice areas than vocal sounds without speech [86]. Yet neuroimaging evidence suggests a general dissociation between speech-related processes (mostly left hemisphere) and speaker identification (mostly right hemisphere) [33,122,157–159]. Thus, the genius of human language could lie both in the interhemispheric dissociation of its components and in the ability to connect spatially segregated regions during communication. Inter-species comparison of the fibre bundles connecting temporal and frontal lobes, such as the arcuate fasciculus, provide useful information: from monkeys to humans, projections are increasingly widespread along the STG and in prefrontal areas [160]. This type of evidence supports the hypothesis that the left dorsal pathway, devoted to the processing of complex sounds in monkeys [131] and to the articulation and production of language in humans [159] has become more complex during the evolution of primates [13,131]. The temporo-parietal regions of the right hemisphere, however, may have evolved in a way that favours multimodal associations and the processing of high-level social information ([148]; see two previous sections). In this scenario, a conserved voice patch system could constitute one of the primitive foundations on which language would have emerged asymmetrically.

## 5. Future directions for comparative research in voice processing

Future work is needed to understand which features drive neuronal responses in the acoustically complex and variable CV and, thus, determine if NHP represent different callers in a measurable ‘voice identity space’ similar to that of humans. The ‘Virtual Monkey’ approach [74] exploiting the natural vocal behaviours of marmosets is a promising technique for this purpose and could be used on new species. A clearer appreciation of the different functional roles of each voice patch will provide crucial information on both an interaction with those of the face-processing system and with language-related areas. In the same line, neuroimaging studies using comparable paradigms across species should also be increasingly conducted to reliably estimate their similarities/differences. Finally, collaborative research in ethology and neuroscience will be essential in the future to improve our knowledge of the environmental and social factors that have influenced the emergence of language and of their respective contributions.

## Data accessibility

This article has no additional data.
